# GSDMD-NETs in patients with sepsis-induced coagulopathy and their interaction with glycocalyx damage

**DOI:** 10.3389/fimmu.2025.1624128

**Published:** 2025-07-22

**Authors:** Xiaoxi Shan, Songmei Yu, Zhaoying Tang, Ping Yang, Lixia Dong

**Affiliations:** ^1^ Department of Respiratory and Critical Care Medicine, Tianjin Medical University General Hospital, Tianjin, China; ^2^ Department of Respiratory and Critical Care Medicine, Yantai Yuhuangding Hospital, Yantai, Shandong, China; ^3^ West Wing Intensive Care Department, Yantai Yuhuangding Hospital, Yantai, Shandong, China; ^4^ Department of Pathology, Yantai Yuhuangding Hospital, Yantai, Shandong, China

**Keywords:** sepsis-induced coagulopathy, neutrophil extracellular traps, gasdermin D, glycocalyx, biomarkers

## Abstract

**Introduction:**

Neutrophil extracellular traps (NETs) play a critical role in inflammation and coagulation imbalance. Recent studies have demonstrated that activation of gasdermin D (GSDMD) protein and its pore-forming activity are essential drivers of NET generation. This study investigated the association between GSDMD-NETs axis activation and sepsis-induced coagulopathy (SIC), as well as the potential association with glycocalyx damage.

**Materials and Methods:**

A prospective cohort of 70 sepsis patients (35 with SIC, 35 non-SIC) admitted to a respiratory intensive care unit was analyzed. This study was registered at the Chinese Clinical Trial Registry (ChiCTR) with the registration number ChiCTR2500100284. The trial can be accessed at https://www.chictr.org.cn/bin/project/edit?pid=266738. Plasma levels of GSDMD-NETs biomarkers (N-GSDMD, MPO-DNA) and glycocalyx injury markers (syndecan-1, MMP-9) were measured via ELISA. Clinical outcomes, thrombotic/hemorrhagic events, and biomarker correlations were evaluated using logistic regression, ROC analysis, and Pearson’s correlation.

**Results:**

Compared to non-SIC patients, the SIC group exhibited higher rates of viral infections (31.4% *vs*. 11.4%, P = 0.043), hemorrhagic events (48.6% *vs*. 17.1%, P = 0.005), and in-hospital mortality (40.0% *vs*. 17.1%, P = 0.034). SIC patients demonstrated significantly elevated GSDMD-NETs axis biomarkers (N-GSDMD: 481.302 *vs*. 539.033, P < 0.001; MPO-DNA: 376.708 *vs*. 461.847, P < 0.001) and glycocalyx damage markers (syndecan-1: 367.754 *vs*. 431.186, P=< 0.001; MMP-9: 121.550 *vs*. 133.931, p = 0.009). GSDMD-NETs biomarkers independently predicted SIC risk (MPO-DNA: OR 1.015, 95% CI 1.005–1.025; N-GSDMD: OR 1.018, 95% CI 1.005–1.031). ROC analysis revealed predictive efficacy for SIC (N-GSDMD: AUC 0.786; MPO-DNA: AUC 0.772), with enhanced performance for their combination (AUC: 0.859). Similarly, the combined biomarkers predicted mechanical ventilation (AUC: 0.755) and mortality (AUC: 0.767). MPO-DNA correlated with syndecan-1 (r = 0.856, p < 0.001) and MMP-9 (r = 0.595, p < 0.001), while N-GSDMD correlated with syndecan-1 (r = 0.343, p = 0.004) and MMP-9 (r = 0.509, p = 0.042).

**Conclusion:**

The activation of the GSDMD-NETs axis is strongly associated with the development of SIC, glycocalyx injury, and adverse clinical outcomes in sepsis, potentially contributing to these pathological processes. Plasma N-GSDMD and MPO-DNA serve as predictive biomarkers for SIC severity and mortality, highlighting their potential role in targeted therapeutic strategies.

## 1 Introduction

Sepsis, a life-threatening dysregulated host response to infection, is characterized by a complex interplay of inflammatory activation and coagulopathic derangements (1). Although physiologic coagulation activation serves as a critical defense mechanism against microbial invasion, its pathological amplification initiates a self-perpetuating cascade of immuno-thrombosis, clotting factor depletion, and subsequent progression to sepsis-induced coagulopathy (SIC). Left unabated, this process culminates in disseminated intravascular coagulation (DIC), multi-organ dysfunction syndrome, and ultimately mortality (2). Despite its clinical significance, the molecular drivers of SIC remain incompletely characterized, limiting targeted therapeutic development.

Emerging mechanistic insights have identified neutrophil extracellular traps (NETs) - chromatin-based antimicrobial structures released through neutrophil death - as central orchestrators of immuno-thrombotic pathology in SIC progression (3, 4). Seminal studies established that GSDMD pore-forming activity, activated through caspase-mediated proteolytic cleavage, serves as an essential prerequisite for NET generation (5, 6). These foundational findings position the GSDMD-NETs axis as a pivotal molecular switch that may be associated with the pathogenesis of SIC. Within the GSDMD - NETs signaling axis, N-terminal gasdermin D (N - GSDMD) acts as the central initiator protein. MPO - DNA complexes serve as a characteristic marker of NETs (7). The activation of the GSDMD - NETs axis can be assessed by measuring the plasma levels of N - GSDMD and MPO - DNA.

Concurrently, the vascular endothelium maintains hemostatic balance through multifaceted regulatory networks, with the endothelial glycocalyx serving as a gatekeeper of thromboresistance, barrier integrity, and inflammatory signaling (8, 9). This dynamic proteoglycan matrix demonstrates marked vulnerability to septic insults, where its degradation not only exacerbates endothelial dysfunction but also establishes a pro-thrombotic milieu permissive for SIC development (10, 11). Specifically, Syndecan - 1, a key component of the glycocalyx, sheds into the bloodstream and results in an elevated plasma concentration, which indicates the damage of the glycocalyx (12). Matrix metalloproteinase - 9 (MMP - 9) is an enzyme involved in glycocalyx degradation, and its increased level is correlated with the extent of glycocalyx injury (13–15).

Building on these insights, this study investigates two interrelated dimensions: (1) the clinical association between GSDMD-mediated NET formation biomarkers and SIC severity, and (2) the possible correlation between GSDMD - NETs axis activation and glycocalyx degradation in septic coagulopathy.

## 2 Materials and methods

### 2.1 Patients and study design

This prospective study, approved by the Ethics Committee of Yantai Yuhuangding Hospital (Approval No. 2023-238) and registered on the Chinese Clinical Trial Registry (ChiCTR2500100284), was conducted from June 2023 to June 2024. The protocol complied with the principles of the Declaration of Helsinki principles and the Ethical Review Measures for Biomedical Research Involving Humans. Written informed consent was obtained from all participants or their legal surrogates prior to enrollment. Consecutively screened adult patients (≥18 years) meeting Sepsis-3 criteria (16) were enrolled within 24 hours of admission to the Respiratory Intensive Care Unit (RICU). SIC classification was determined using the 2017 ISTH criteria (17), requiring concurrent assessment of platelet count, prothrombin time, and SOFA score during the initial 24-hour observation window. Exclusion criteria included: 1) death or discharge against medical advice within 24 hours post-enrollment; 2) pre-admission diagnosis of overt DIC; 3) age <18 years; 4) non-consenting status. All patients received protocolized care according to the 2021 Surviving Sepsis Campaign Guidelines (18), including antimicrobial stewardship, hemodynamic monitoring, and organ support therapies.

**Figure 1:**
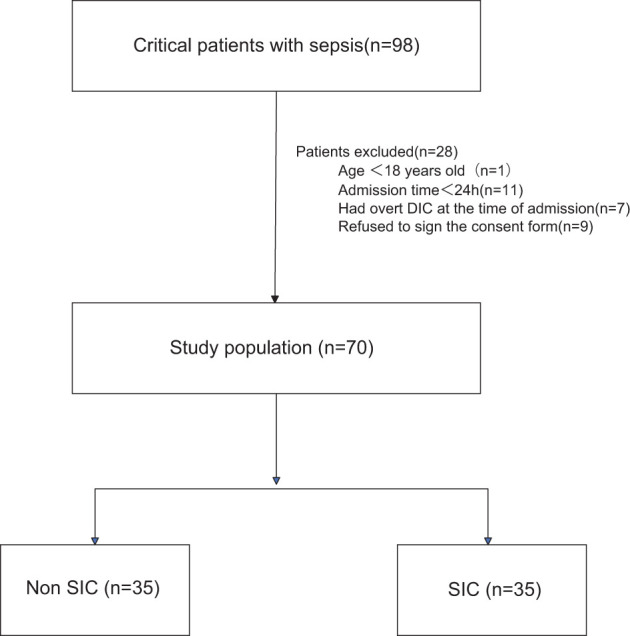
Flowchart of the patients included in the study. DIC, disseminated intravascular coagulation; SIC, Sepsis - Induced Coagulopathy.

During the study period, a total of 98 patients were admitted with a diagnosis of sepsis. Among them, 1 patient was excluded for being under 18 years old, 11 patients died within 24 hours after admission, and 7 patients were excluded for having overt disseminated intravascular coagulation (DIC) at the time of admission. Additionally, 9 patients refused to sign the informed consent form. Therefore, a total of 70 patients were included in our study, of whom 35 met the criteria for SIC (**Figure 1**).

### 2.2 Collection of clinical data and outcomes

Within 24 hours of RICU admission, comprehensive clinical characterization was performed including structured history-taking, systematic laboratory profiling, and quantification of plasma biomarkers reflecting GSDMD-NETs axis activation and glycocalyx integrity. Standardized data collection encompassed demographics (age/sex), comorbidities, inflammatory markers (procalcitonin, CRP, neutrophil/lymphocyte counts), nutritional status (albumin), and coagulation parameters (D-dimer). Thrombotic events were defined as radiologically confirmed deep-vein thrombosis, pulmonary embolism, or acute arterial thrombosis (coronary/cerebral/peripheral). Hemorrhagic complications included endoscopically/proven gastrointestinal bleeding, imaging-confirmed intracranial hemorrhage, or macroscopic hematuria. Mechanical ventilation requirement and in-hospital mortality were tracked as clinical endpoints.

### 2.3 Sample collection and ELISA assay operations

Within 24 hours after admission, venous blood samples were collected into ethylenediaminetetraacetic acid (EDTA) tubes and processed within 30 minutes (3000 g for 15 minutes at 4°C). The samples were then aliquoted and stored at -80°C until batch analysis.

According to the manufacturer’s instructions, the levels of serum MPO - DNA, N - GSDMD, MMP - 9, and Syndecan - 1 were measured using commercial ELISA kits (Jiangsu Meimian Industrial Co., Ltd). The specific kits used were the Human MPO - DNA ELISA kit (Catalog No.: MM - 2467H1), Human N - GSDMD ELISA kit (Catalog No.: MM - 51628H1), Human MMP - 9 ELISA kit (Catalog No.: MM - 0149H1), and Human Syndecan - 1 ELISA kit (Catalog No.: MM - 1818H1). The operations were carried out strictly following the manufacturer’s protocols.

The provided microplates were pre - coated with corresponding specific antibodies. Standards and samples were added to the appropriate wells of the microplate, with three replicate wells set for each standard or sample. Subsequently, the corresponding biotin - conjugated antibodies were added. A color change occurred because of the reaction between the biotin - conjugated antibodies and the enzyme - conjugated avidin. The enzyme - substrate reaction was terminated by adding sulfuric acid solution, and then the color change was measured at a wavelength of 450 nm using a spectrophotometer. A standard curve was plotted by measuring the absorbance and concentration of the standards. The concentration of the samples was determined by comparing the absorbance values of the samples with the standard curve.

### 2.4 Statistical analysis

Continuous variables adhering to a normal distribution were evaluated with the independent samples t-test, with outcomes reported as mean ± standard deviation. For datasets violating normality assumptions, nonparametric comparisons were performed using the Wilcoxon rank-sum test (Mann-Whitney U equivalent), and results are summarized using median values with interquartile ranges. Dichotomous and categorical parameters underwent comparative analysis through Pearson’s chi-square test or Fisher’s exact probability test where appropriate, with frequency distributions expressed as percentages. A multivariable logistic regression model was constructed to assess potential predictors associated with SIC risk stratification. The predictive performance of plasma GSDMD-NETs biomarker levels for SIC, in-hospital mortality, and mechanical ventilation requirement was evaluated through receiver operating characteristic (ROC) curve analysis. The association between plasma levels of GSDMD-NETs biomarkers (e.g., N-GSDMD, MPO-DNA) and glycocalyx injury markers (e.g., syndecan-1, MMP-9) was analyzed using Pearson’s correlation coefficient. Data analyses were conducted with IBM SPSS Statistics (Version 13.0; SPSS Inc., Chicago, IL, USA) for general statistical procedures and MedCalc (Version 14.8; MedCalc Software Ltd., Ostend, Belgium) for receiver operating characteristic (ROC) curve computations. Statistical significance was defined as a two-sided *P*<0.05.

## 3 Result

### 3.1 Baseline clinical characteristics of the study cohort

A total of 70 sepsis patients were included, 35 with SIC and 35 without SIC. **Table 1** presents the baseline characteristics of sepsis patients after ICU admission across different groups. No significant differences were identified between the groups in age or sex distribution. The prevalence of comorbidities, including chronic pulmonary, cardiovascular, cerebrovascular, renal, hepatic diseases, diabetes, and active malignancy, also showed no significant differences between the groups. In terms of pathogens, the distribution was similar except for a higher proportion of viral infections in the SIC group (p = 0.043). Laboratory examinations revealed that the neutrophil-to-lymphocyte ratio (NLR) was significantly higher in the SIC group compared to the non-SIC group (p = 0.040). While levels of procalcitonin (PCT) and C-reactive protein (CRP) were higher in the SIC group, these differences did not reach statistical significance. Similarly, D-dimer levels were elevated in the SIC group but also did not reach statistical significance.

**Table 1:** Baseline characteristics of sepsis patients in different groups.

Characteristics	Non-SIC, n=35	SIC, n=35	P
Age,year	71 (64,79)	71 (65,81)	0.652
Sex,n (%)			
Male	22 (62.9)	22 (62.9)	1.000
Co-morbidities,n (%)			
Chronic pulmonary diseases	15 (42.9)	16 (45.7)	0.810
Chronic cardiovascular diseases	25 (71.4)	24 (68.6)	0.794
Chronic cerebrovascular diseases	14 (40.0)	13 (37.1)	0.806
Chronic renal diseases	4 (11.4)	5 (14.3)	0.721
Chronic hepatic disease	6 (17.1)	5 (14.3)	0.743
Diabetes	11 (31.4)	11 (31.4)	1.000
Active Malignancy	5 (14.3)	6 (17.1)	0.743
Pathogen,n (%)			
Gram-positive bacteria	10 (28.6)	6 (17.1)	0.255
Gram-negative bacteria	18 (51.4)	15 (42.9)	0.473
Fungus	3 (8.6)	5 (14.3)	0.452
Virus	4 (11.4)	11 (31.4)	0.043
Laboratory Examinations			
PCT	0.568 (0.114,1.130)	0.644 (0.200,3.950)	0.315
CRP	89.610 (18.103,147.680)	107.610 (40.600,185.000)	0.470
Albumin	31.350 (28.590,34.791)	30.620 (26.470,33.570)	0.136
Neutrophil-to-Lymphocyte Ratio	9.820 (5.547,9.820)	15.413 (9.767,26.431)	0.040
D dimer (mg/L)	3.130 (1.860,8.020)	4.590 (1.990,10.270)	0.333

SIC, Sepsis - Induced Coagulopathy; PCT, Procalcitonin; CRP, C-reactive protein.

**Figure 2:**
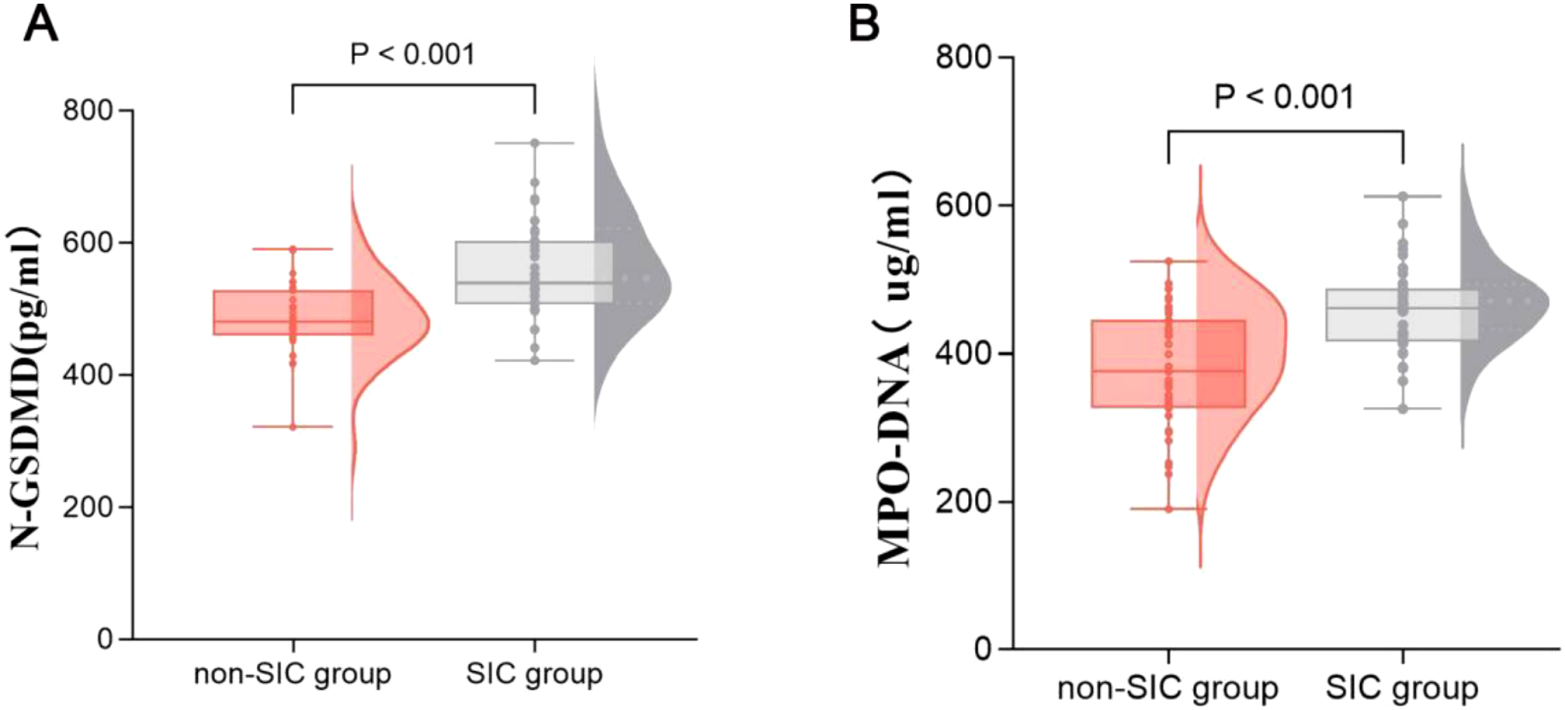
Comparison of GSDMD-NETs axis markers, including N-GSDMD **(A)** and MPO-DNA **(B)** in groups of patients with or without SIC. SIC, Sepsis - Induced Coagulopathy; GSDMD, Gasdermin D; NETs, Neutrophil Extracellular Traps.

**Table 2** presents the GSDMD-NETs axis markers and clinical outcomes in sepsis patients across different groups. The levels of MPO-DNA [376.708 (325.886–445.340) *vs*. 461.847 (416.237–487.910), p < 0.001] and N-GSDMD [481.302 (459.549–528.518) *vs*. 539.033 (507.330–602.978), p < 0.001] were significantly higher in the SIC group compared to the non-SIC group, as shown in **Figure 2**. In terms of clinical outcomes, the SIC group had higher in-hospital mortality (40.0% *vs*. 17.1%, p = 0.034) and mechanical ventilation rates (58.1% *vs*. 28.6%, p = 0.029). Thrombotic events showed no significant difference between the groups (45.7% *vs*. 51.4%, p = 0.632), while hemorrhagic events were significantly higher in the SIC group (48.6% *vs*. 17.1%, p = 0.005).

**Table 2:** GSDMD-NETs axis marker and outcomes in sepsis patients in different groups.

Characteristics	Non-SIC, n=35	SIC, n=35	P
GSDMD-NETs axis marker			
MPO-DNA (μg/ml)	376.708 (325.886,445.340)	461.847 (416.237,487.910)	<0.001
N-GSDMD (pg/ml)	481.302 (459.549,528.518)	539.033 (507.330,602.978)	<0.001
Outcome			
In-hospital mortality	6 (17.1)	14 (40.0)	0.034
Mechanical ventilation	10 (28.6)	20 (58.1)	0.029
Thrombotic events	18 (51.4)	16 (45.7)	0.632
Hemorrhagic events	6 (17.1)	17 (48.6)	0.005

SIC, Sepsis - Induced Coagulopathy; GSDMD, Gasdermin D; NETs, Neutrophil Extracellular Traps.

### 3.2 Independent risk factors for sepsis-induced coagulopathy

To identify variables associated with SIC in sepsis patients, a univariate logistic regression analysis was performed. The analysis revealed that Pathogen (Virus), Neutrophil-to-Lymphocyte Ratio, MPO-DNA, and N-GSDMD were associated with SIC. Therefore, these variables were taken into multivariate logistic regression for adjustment. The OR for SIC of MPO-DNA was 1.015 (95% CI, 1.005–1.025, p = 0.003), and N-GSDMD was 1.018 (95% CI, 1.005–1.031, p = 0.006) after adjustment for risk factors (**Tables 3**, **4**).

**Table 3:** Univariate logistic regression analysis for possible risk factors for SIC.

Variable	B	SE	p	OR	95% CI for OR	
					Lower	Upper
Pathogen, Virus	1.268	0.644	0.049	3.552	1.005	12.552
Neutrophil-to-Lymphocyte Ratio	0.039	0.02	0.048	1.039	1.001	1.082
MPO-DNA (μg/ml)	0.017	0.005	<0.001	1.017	1.008	1.026
N-GSDMD (pg/ml)	0.021	0.006	<0.001	1.021	1.009	1.033

GSDMD, Gasdermin D; SIC, Sepsis - Induced Coagulopathy.

**Table 4:** Multivariate logistic regression analysis for possible risk factors for SIC.

Variable	B	SE	p	OR	95% CI for OR	
					Lower	Upper
Pathogen, Virus	1.549	0.932	0.097	4.705	0.757	29.249
Neutrophil-to-Lymphocyte Ratio	0.044	0.027	0.107	1.045	0.991	1.102
MPO-DNA (μg/ml)	0.015	0.005	0.003	1.015	1.005	1.025
N-GSDMD (pg/ml)	0.018	0.006	0.006	1.018	1.005	1.031

GSDMD, Gasdermin D; SIC, Sepsis - Induced Coagulopathy.

**Figure 3:**
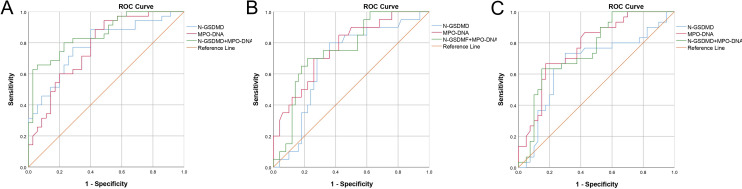
Receiver operating characteristic (ROC) curve of N-GSDMD, MPO-DNA and combination of N-GSDMD and MPO-DNA for prediction of **(A)** SIC, **(B)** In-hospital mortality and **(C)** mechanical ventilation. SIC, Sepsis - Induced Coagulopathy.

An ROC curve for SIC occurrence was drawn (**Figure 3A**). As shown in **Table 5**, the area under the curve for N-GSDMD was 0.786 (95% CI, 0.679–0.893) with a sensitivity of 0.886 and a specificity of 0.6. The area under the curve for MPO-DNA was 0.772 (95% CI, 0.663–0.881) with a sensitivity of 0.943 and a specificity of 0.514. The combination of N-GSDMD and MPO-DNA showed a statistically significant improvement in predictive efficacy compared to N-GSDMD alone (p = 0.0480) and MPO-DNA alone (p = 0.0475). The area under the curve for the combination was 0.859 (95% CI, 0.773–0.944), indicating a more robust predictive value for SIC.

**Table 5:** Receiver operating characteristic curve analysis of N-GSDMD, MPO-DNA, RDW, combination of N-GSDMD and MPO-DNA.

Outcomes	Index	AUC	95% CI	P-value*	Sensitivity	Specificity
SIC	N-GSDMD	0.786	0.679-0.893	0.048	0.886	0.600
	MPO-DNA	0.772	0.663-0.881	0.0475	0.943	0.514
	combination of N-GSDMD and MPO-DNA	0.859	0.773-0.944		0.657	0.943
In-hospital mortality	N-GSDMD	0.683	0.549-0.817	0.0886	0.700	0.740
	MPO-DNA	0.767	0.648-0.886	0.8396	0.800	0.640
	combination of N-GSDMD and MPO-DNA	0.756	0.637-0.875		0.700	0.780
Mechanical ventilation	N-GSDMD	0.667	0.532-0.802	0.0321	0.667	0.825
	MPO-DNA	0.773	0.663-0.882	0.7345	0.733	0.700
	combination of N-GSDMD and MPO-DNA	0.755	0.641-0.869		0.630	0.850

SIC, Sepsis - Induced Coagulopathy; GSDMD, Gasdermin D. P-value *:Compared with the AUC of combination of N-GSDMD and MPO-DNA.

An ROC curve for SIC occurrence was drawn (**Figure 3A**). As shown in **Table 5**, the area under the curve for N-GSDMD was 0.786 (95% CI, 0.679–0.893) with a sensitivity of 0.886 and a specificity of 0.6. The area under the curve for MPO-DNA was 0.772 (95% CI, 0.663–0.881) with a sensitivity of 0.943 and a specificity of 0.514. The combination of N-GSDMD and MPO-DNA showed a statistically significant improvement in predictive efficacy compared to N-GSDMD alone (p = 0.0480) and MPO-DNA alone (p = 0.0475). The area under the curve for the combination was 0.859 (95% CI, 0.773–0.944), indicating a more robust predictive value for SIC.

### 3.3 Predictive capabilities of GSDMD-NETs axis markers for sepsis patients outcomes

Using ROC curves, the predictive capabilities of N-GSDMD and MPO-DNA for in-hospital mortality and mechanical ventilation were examined (**Figures 3B, C**). As **Table 5** shown, for in-hospital mortality, the AUC for N-GSDMD was 0.683 (95% CI, 0.549–0.817) with a sensitivity of 0.700 and a specificity of 0.740. The AUC for MPO-DNA was 0.767 (95% CI, 0.648–0.886) with a sensitivity of 0.800 and a specificity of 0.640. In contrast to the prediction of SIC, the combination of N-GSDMD and MPO-DNA did not enhance the predictive efficacy for in-hospital mortality. For mechanical ventilation, the AUC for N-GSDMD was 0.667 (95% CI, 0.532–0.802) with a sensitivity of 0.667 and a specificity of 0.825. The AUC for MPO-DNA was 0.773 (95% CI, 0.663–0.882) with a sensitivity of 0.733 and a specificity of 0.700. The combination of N-GSDMD and MPO-DNA showed a higher predictive efficacy, with an AUC of 0.755 (95% CI, 0.641–0.869) and a sensitivity of 0.630 and a specificity of 0.850.

### 3.4 Glycocalyx biomarkers in the sepsis - induced coagulopathy group

**Figure 4:**
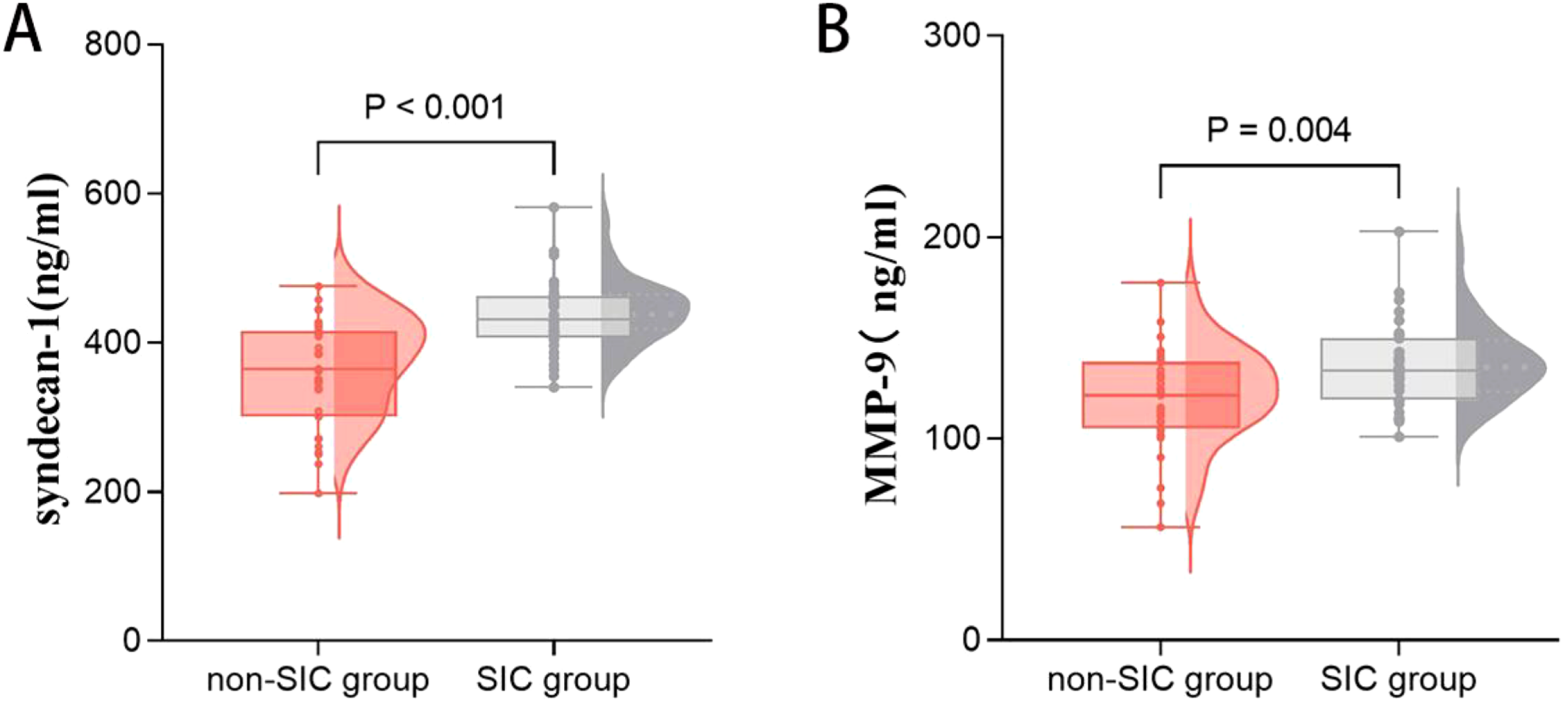
Comparison of glycocalyx axis markers, including syndecan-1 **(A)** and MMP-9 **(B)** in groups of patients with or without SIC. SIC, Sepsis - Induced Coagulopathy; MMP-9, Matrix Metalloproteinase-9.

Biomarkers of the glycocalyx were also examined. Syndecan-1 [367.754 (300.579–416.171) *vs*. 431.186 (407.071–463.492), p < 0.001] was significantly higher in the SIC group compared to the non-SIC group. Similarly, MMP-9 [121.550 (104.902–138.334) *vs*. 133.931 (119.348–149.891), p = 0.009] levels were also higher in the SIC group (shown in **Table 6**, **Figure 4**).

**Table 6:** Levels of biomarkers of glycocalyx in different groups.

Characteristics	Non-SIC,n=35	SIC,n=35	P
syndecan-1 (ng/mL)	367.754 (300.579, 416.171)	431.186 (407.071, 463.492)	<0.001
MMP-9 (ng/mL)	121.550 (104.902, 138.334)	133.931 (119.348, 149.891)	0.009

SIC, Sepsis - Induced Coagulopathy; MMP-9, Matrix Metalloproteinase-9.

### 3.5 Relationship between GSDMD-NETs axis markers and glycocalyx biomarkers

**Figure 5:**
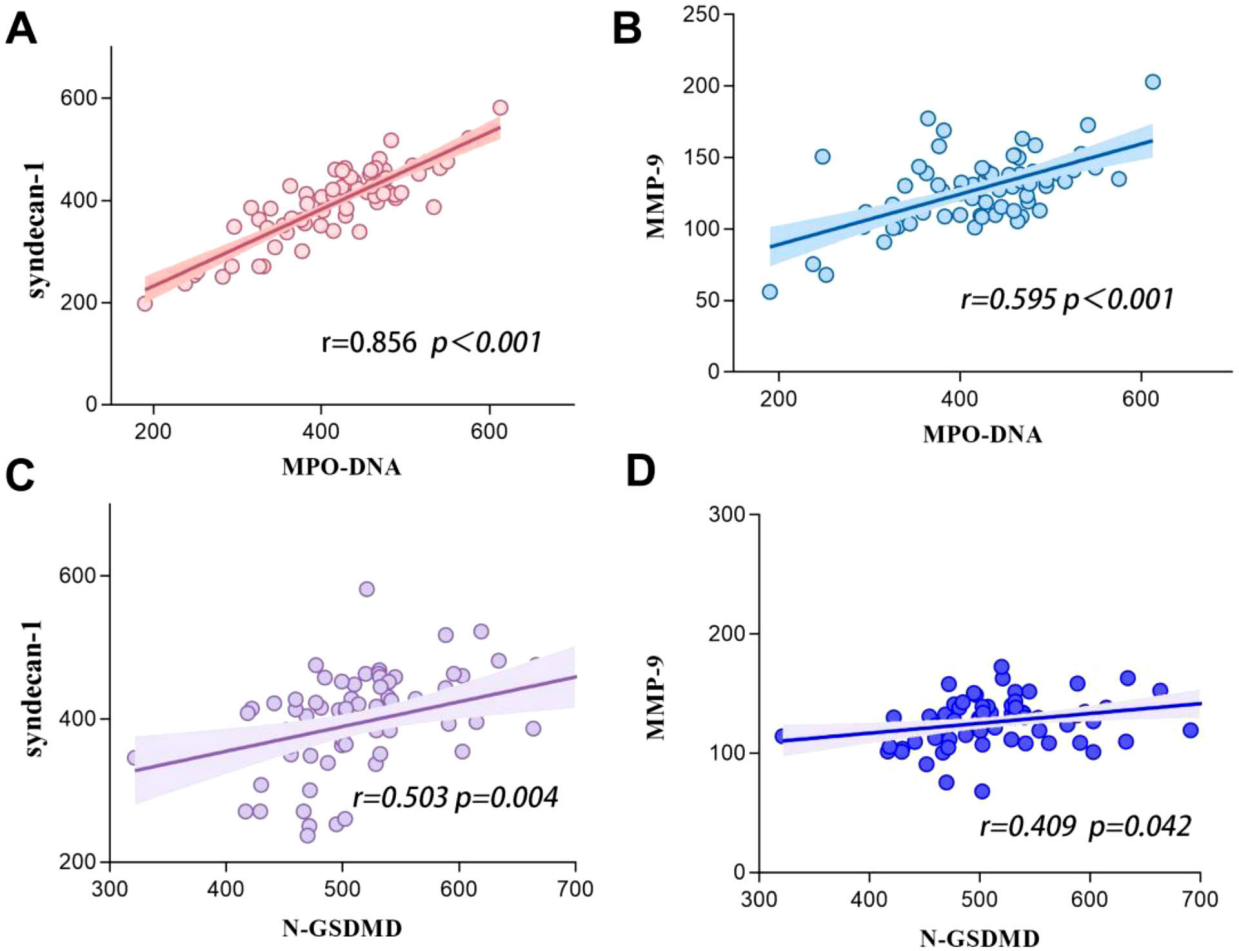
Correlations between GSDMD-NETs axis markers level (including N-GSDMD and MPO-DNA) and glycocalyx biomarkers (including syndecan-1 and MMP-9) in patients with sepsis. **(A)** Syndecan-1 vs MPO-DNA. **(B)** MMP-9 vs MPO-DNA. **(C)** Syndecan-1 vs N-GSDMD. **(D)** MMP-9 vs N-GSDMD.

We hypothesized that the GSDMD-NETs axis interacts closely with glycan damage in the development of SIC. Pearson analysis revealed significant correlations between MPO-DNA and syndecan-1 (r = 0.856, p < 0.001), between MPO-DNA and MMP-9 (r = 0.595, p < 0.001), between N-GSDMD and syndecan-1 (r = 0.343, p = 0.004), and between N-GSDMD and MMP-9 (r = 0.509, p = 0.042)(**Figure 5**).

## 4 Discussion

This study provides the first human evidence specifically regarding the activation of the systemic GSDMD - NETs axis within the pathophysiology of SIC. To delineate the pathophysiological role of this axis in septic coagulopathy, we quantified plasma N-GSDMD (executioner of pyroptotic NETosis) and MPO-DNA (hallmark NET complexes), revealing marked elevations in SIC patients versus non-SIC controls: MPO-DNA [median (IQR): 461.847 (416.237–487.910) *vs*. 376.708 (325.89–445.34), P<0.001]; N-GSDMD [539.033 (507.33–602.98) *vs*. 481.302 (459.55–528.52), P<0.001]. Mechanistically, GSDMD-NETs activation emerged as an independent SIC predictor (MPO-DNA: OR 1.015, 95%CI 1.005–1.025; N-GSDMD: OR 1.018, 95%CI 1.005–1.031). Clinically, ROC analysis positioned these biomarkers as sensitive stratifiers for SIC development (AUC: 0.772 [MPO-DNA] *vs*. 0.786 [N-GSDMD]; combined AUC 0.859), mechanical ventilation requirement (AUC 0.718–0.742), and mortality risk (AUC 0.753–0.769). Notably, SIC patients exhibited virological predisposition (31.4% viral infections), neutrophil-dominant inflammation (elevated NLR), and hemorrhagic diathesis (48.6% bleeding events). These findings collectively establish SIC as a distinct immunothrombotic phenotype with accelerated organ dysfunction trajectories.

To our knowledge, this study represents the first prospective clinical investigation that focuses on the activation of the GSDMD - NETs axis in sepsis patients who develop SIC. With evolving understanding of sepsis pathophysiology, accumulating evidence highlights the interplay between immune dysregulation and coagulation disturbances in driving immunothrombosis, thereby contributing to SIC development. The emergence of SIC frequently portends unfavorable clinical outcomes. A study of 1,895 Japanese ICU patients demonstrated that 29% of sepsis patients met SIC diagnostic criteria (19). A large-scale analysis of 9,432 sepsis cases further confirmed SIC as an independent risk factor for 7- and 28-day mortality (adjusted OR=1.52), with distinct survival curve separation between SIC and non-SIC cohorts (20). These findings underscore the critical importance of early SIC identification and intervention in sepsis management. Neutrophils, the most rapidly recruited and abundant immune cells in sepsis, undergo cytoskeletal reorganization during sepsis progression to release neutrophil extracellular traps (NETs)—web-like structures that entrap pathogens (21). Recent clinical studies have increasingly elucidated the intricate relationship between NETs and coagulation activation (22–24), positioning NETs as a promising therapeutic target for SIC. Building upon clinical evidence demonstrating that SARS-CoV-2 virus directly activates the GSDMD pathway to trigger NET release and mediate organ damage in COVID-19 patients (25), where analysis of blood and lung tissue samples from hospitalized patients revealed GSDMD expression and NET formation associated with disease severity. Emerging preclinical evidence from murine sepsis models reveals that Gasdermin D (GSDMD) generates its N-terminal domain (N-GSDMD), which facilitates NET release through plasma membrane pore formation. Genetic ablation of *GSDMD* or pharmacological inhibition (e.g., LDC7559) markedly reduces NET generation and prolongs survival in septic mice, identifying the GSDMD-NETs axis as a pivotal regulatory node (5, 6).These mechanistic insights not only deepen our interest in this pathway but also prompt our hypothesis: biomarkers of GSDMD-NETs axis activation may hold significant clinical relevance in sepsis patients with SIC. In this study, for the first time in the specific context of SIC, we demonstrate that sepsis patients with SIC show significantly elevated plasma levels of GSDMD - NETs signature proteins (N - GSDMD and MPO - DNA), and these elevations independently correlate with the risk of SIC. Serum concentrations of these biomarkers effectively predict disseminated intravascular coagulation (DIC), mechanical ventilation requirement, and in-hospital mortality during sepsis. These findings provide clinical validation for the pathophysiological significance of the GSDMD-NETs axis in SIC progression.

At the cellular level, vascular endothelial cells play a pivotal role in maintaining hemostatic balance (26). The endothelial glycocalyx, composed of proteoglycans and glycosaminoglycans, preserves vascular homeostasis through electrostatic repulsion, anticoagulant molecule adsorption, and intercellular junction stabilization (27). However, this fragile structure is susceptible to degradation during sepsis. Syndecan-1, a core proteoglycan component of the glycocalyx, sheds into circulation upon glycocalyx injury (8, 9). Lipopolysaccharides (LPS) further exacerbate this damage by activating matrix metalloproteinase-9 (MMP-9) via Toll-like receptor 4 (TLR4), directly degrading glycocalyx components, exposing subendothelial collagen, and inducing tissue factor expression to establish a procoagulant microenvironment (10, 11). Prior studies have consistently linked elevated syndecan-1 and MMP-9 levels to adverse outcomes and multi-organ dysfunction in sepsis (28–30). Our findings align with this evidence, demonstrating significantly higher circulating MMP-9 and syndecan-1 levels in SIC patients compared to non-SIC counterparts, indicative of more severe glycocalyx disruption (24). Intriguingly, our data reveal a robust correlation between GSDMD-NETs axis activation and glycocalyx injury biomarkers: MPO-DNA exhibited strong associations with syndecan-1 (r = 0.856, p < 0.001) and MMP-9 (r = 0.595, p < 0.001), while N-GSDMD correlated moderately with syndecan-1 (r = 0.503, p = 0.004) and MMP-9 (r = 0.409, p = 0.042). This parallels prior evidence highlighting neutrophil-endothelial interactions as critical drivers of coagulopathy in sepsis (31). Notably, while GSDMD-NETs have been implicated in thrombosis during non-inflammatory conditions such as sickle cell anemia (32), their role in SIC pathogenesis remains underexplored. Our study provides novel clinical evidence supporting the hypothesis that GSDMD-NETs axis activation may be involved in SIC progression through glycocalyx destabilization.

Our study yielded several intriguing clinical observations. Compared to the non-SIC group, a higher proportion of SIC patients developed sepsis secondary to viral infections, suggesting that viruses may predispose to SIC more readily than bacterial pathogens. This aligns with post-COVID-19 pandemic findings, where autopsy studies of fatal COVID-19 cases revealed widespread thrombotic events and vascular endothelial injury (33), underscoring the need for heightened vigilance against SIC in viral sepsis management. Notably, while the incidence of thrombotic events did not significantly differ between SIC and non-SIC groups (51.4% *vs*. 45.7%, P=0.632), the SIC cohort exhibited a markedly elevated risk of hemorrhagic complications (48.6% *vs*. 17.1%). Based on current guidelines (34), we advocate initiating anticoagulation therapy upon SIC diagnosis. However, clinicians must remain cautious: the concurrent depletion of coagulation substrates, thrombocytopenia, and immunothrombosis in SIC patients substantially amplifies bleeding risks. Close monitoring for hemorrhagic manifestations is critical during treatment. These findings should be interpreted with caution due to our study’s limited sample size, necessitating validation through larger-scale clinical investigations to clarify the interplay between viral etiology, SIC pathophysiology, and anticoagulation-associated bleeding risks.

This study has several limitations. Firstly, the single-center design, relatively small sample size, and inability to conduct subgroup analyses based on pathogen types (e.g., bacterial, viral, fungal infections) may constrain the generalizability of findings and limit insights into pathogen-specific heterogeneity in GSDMD-NETs axis activation among sepsis patients. Future multi-center studies with larger cohorts are needed to validate these findings and explore such heterogeneity. Secondly, in this study, we mainly used the ELISA to measure the markers of the GSDMD - NETs axis (N - GSDMD, MPO - DNA) and the markers of glycocalyx damage (syndecan - 1, MMP - 9), and analyzed their correlations. The detection method was relatively single, and no other methods were used for verification. For example, zymography could be used to measure the activity of MMP - 9, and the release of NETs could be quantified by immunofluorescence after isolating neutrophils. More experimental methods are needed for verification in the future. Thirdly, although GSDMD-NETs biomarker levels were measured within 24 hours of admission, longitudinal monitoring during treatment was lacking, leaving the temporal dynamics of this pathway unexplored. Future investigations incorporating serial sampling at multiple time points may clarify the evolving relationship between GSDMD-NETs axis activation, coagulation disturbances, and organ dysfunction, thereby informing targeted therapeutic strategies.

## 5 Conclusions

Elevated levels of GSDMD-NETs axis biomarkers (N-GSDMD and MPO-DNA) were significantly associated with the incidence of SIC, mechanical ventilation requirement, and in-hospital mortality in sepsis patients, demonstrating a significant relationship with glycocalyx damage.

## Data Availability

The original contributions presented in the study are included in the article/supplementary material. Further inquiries can be directed to the corresponding author.
